# Digital transformation, green finance, and pharmaceutical affordability in China: a health economics perspective

**DOI:** 10.3389/fpubh.2026.1793373

**Published:** 2026-05-15

**Authors:** Jingdong Huang, Xinyi Zhang

**Affiliations:** School of Finance and Economics, Tibet University, Lhasa, China

**Keywords:** digital transformation, drug accessibility, essential drugs, financing costs, green finance, health equity

## Abstract

**Introduction:**

Drug accessibility is a core challenge facing global public health systems, with financing costs directly affecting drug prices through cost-plus pricing mechanisms. This study aims to examine how digital transformation influences pharmaceutical companies' financing costs by promoting green certification and green finance availability, and to explore the heterogeneous effects of essential drug enterprises and their public health significance.

**Methods:**

Based on 1,216 observations from 152 A-share listed pharmaceutical manufacturing companies in China from 2016–2023, this study employs fixed-effects panel data models to test the impact of digital transformation on financing costs, uses Bootstrap methods to test chain mediation effects, and controls for endogeneity through instrumental variable methods and propensity score matching-difference-in-differences approaches.

**Results:**

Digital transformation significantly increases the probability of enterprises obtaining green certification (β = 0.079, *p* < 0.01), green certification significantly promotes green finance availability (β = 0.134, *p* < 0.01), and green finance significantly reduces financing costs by approximately 48.7 basis points (β = −0.487, *p* < 0.01). Mediation effect analysis shows that the chain mediation effect accounts for 34.2% of the total effect. The effect of essential drug enterprises reducing financing costs through digital transformation is 1.9 times that of non-essential drug enterprises (−0.051 vs. −0.027, *p* < 0.05). Mechanism testing indicates that information asymmetry mitigation and risk premium reduction explain 39% and 53% of the total effect, respectively.

**Discussion:**

Digital transformation significantly reduces pharmaceutical companies' financing costs through the chain mediation of green certification and green finance, with the effect being more pronounced for essential drug enterprises. The reduction in financing costs may create room for price adjustment. Under China's essential drug price regulation system, cost savings could potentially be transmitted to essential drug prices, which has theoretical implications for improving drug accessibility and promoting health equity. It is recommended to improve the green finance standard system, strengthen differentiated policy support for essential drug enterprises, and establish a linkage mechanism between financing costs and drug prices.

## 1 Introduction

Drug accessibility is a key challenge facing global health systems. Statistics by the World Health Organization show that every year, 100 million people worldwide fall into extreme poverty due to catastrophic health spending, of which drug spending has been identified as a key impoverishing factor (1). This complexity arises because drug pricing models reflect the aggregated costs incurred across different stages, including R&D, manufacturing, and distribution. Financing costs are an important part of business operational costs for pharmaceutical companies and are incorporated in the final prices of drugs as part of cost-plus pricing (2). The pharmaceutical industry, as a typical capital-intensive sector, has R&D cycles exceeding 10 years and requires heavy investment. Consequently, pharmaceutical companies depend on external financing to a considerably greater extent than firms in other manufacturing sectors and are highly sensitive to changes in financing costs (3). In China, progressive reforms in the essential drug scheme and national procurement policies have achieved notable outcomes, including substantial price reductions for certain drugs (4). However, structural challenges remain, such as underinvestment in innovative drug R&D and continued dependence on imported advanced formulations. The financing constraints facing pharmaceutical corporations have adversely affected industrial upgrading and the improvement of drug supply capacity. To improve drug availability, it is imperative to address both the profit margins in the distribution sector as well as the financing costs of enterprises (5, 6).

China has improved its institutional innovation on green finance and has developed new policy tools that can tackle the aforementioned problems. In 2016, the People's Bank of China and eight other ministries issued the “Guiding Opinions on the Establishment of a Green Financial System,” formally initiating the systematic promotion of green finance development. China's balance of green loans exceeded 30 trillion yuan at the end of 2023, and it had the second-largest amount of green bonds in the world. The use of diversified financial tools such as the setting up of green development funds, green bonds, and green loans channels funds into industries that are environmentally sustainable (7). Green finance reform and innovation pilot zones were introduced in five provinces and regions in 2017, and this has created experience for replication at a national level. The pharmaceutical industry has traditionally been considered highly polluting and energy-intensive, and now faces growing pressure for green transformation due to stricter environmental regulations. However, the industry also has strong potential for policy support given the public health attributes of its products (8). The pharmaceutical companies that acquire green certification can benefit from policy advantages, such as favorable costs for issuing green bonds (market practice shows an average of 30–60 basis points lower), reduced green credit risk weights, and fiscal interest subsidies. These new channels will help pharmaceutical companies reduce financing costs.

Meanwhile, the digital transformation is increasingly penetrating different industries. New technologies such as big data, artificial intelligence, and blockchain technology have shown great potential for improving the efficiency of operations and the transparency of information for enterprises, thus providing a way for the green transformation of traditional industries (9). Digital technologies allow enterprises to track their environmental performance in real time at a lower cost. Technology applications such as the Internet of Things, blockchain, and artificial intelligence make information costs lower in acquiring green certification. These technology applications lower the information and time costs of acquiring third-party green certification, providing pharmaceutical companies with a feasible pathway to reduce financing costs through green finance. More importantly, due to their products' public health attributes, the essential drug manufacturing enterprises may enjoy high priority support in the green finance policies. If the above assumption is correct, the decrease in the cost of financing brought about by the essential drug enterprises‘ digital green transformation may, under certain institutional conditions, be more likely to pass on to essential drug prices, potentially benefiting impoverished communities in achieving health equity.

Existing literature has played a part in offering a relatively complete understanding of the micro-level business impacts of green finance. Green bonds, under the framework of green finance, are a crucial tool for transmitting genuine investment intentions of firms regarding the environment to capital markets via signaling mechanisms to offset information asymmetry (10). Assessments of the policy effects of the pilot policies of China's green finance pilot zones show that the pilot policies have caused a substantial increase in the market value of green firms in the regions, through two mechanisms including capital market effects and operational effects, which confirm the role of policy-based financial support in the promotion of enterprise green transformation (11). The effect of green finance on debt financing for enterprises shows asymmetrical patterns, which are heterogeneous regarding the types of enterprises in deriving benefits from policies. This is observed in state-owned vs. other private enterprises, as well as large vs. small and medium-sized enterprises (12).

The role of digital transformation in promoting enterprise Environmental, Social, and Governance (ESG) performance has gradually emerged as an important research topic. Digital technology plays its role in enhancing the performance of the enterprise in terms of the mentioned variables through various mechanisms, which include the improvement of the quality of internal control, increased transparency of information, and the enhancement of innovation capability (9). The digital transformation of manufacturing enterprises not only helps to optimize production processes and lower energy consumption but also enhances trust among various stakeholders by implementing traceable supply chain management systems, thus providing favorable conditions for enterprises to gain support for green finance (13). That the positive correlation between the level of digitalization of enterprises and their ESG performance has been proved in different industries; however, there are significant differences in terms of the applicability of digital technologies and improvement in ESG in different industries (14).

Studies on green supply chain management in the pharmaceutical industry show that green transformation helps in lowering operating costs, but the high initial investment cost deters the willingness of the firm to undergo the transformation (15). Research on digital transformation in the pharmaceutical industry has gradually expanded to cover areas such as intelligent manufacturing, precision medicine, and drug traceability. Nevertheless, the current literature is found to be limited in exploring the effect of digitalization on corporate financing decisions and financial performance (16). Regarding the relationship between digital transformation in pharmaceutical enterprises and healthcare equity, there is some preliminary evidence of a positive relationship between digital technology usage and improved transparency in terms of finances in pharmaceutical enterprises. The processes by which improved transparency leads to improved access to medications by end-users remain unclear (17).

Although the above studies explore the relationships among green finance, digital transformation, and enterprise performance from different perspectives, existing literature has three research gaps:

First, there is an absence of an overarching process by which the promotion of green finance through digital transformation has been explained. The existing literature has already shown that digital transformation improves ESG outcomes (9, 13, 14) and that green certification reduces financing costs (10, 11), but there is still an absence of empirical examination of the complete process by which digital transformation leads to green certification acquisition, eventually reducing financing costs. Particularly, the transmission mechanisms and relative contributions of each link in the chain mediation path of “digital transformation → green certification → green finance → financing costs” remain unclear.

Second, as a special industry combining high R&D investment with public health attributes, the pharmaceutical manufacturing sector's transmission channel from financing cost volatility to the availability of medications has not been adequately investigated. However, existing studies on green finance have been focusing more on financial performance for enterprises (such as Tobin's Q, stock returns, credit rating improvements, etc.), without any relevant examination of the issue of lower costs of financing and its effects of improving patient accessibility to medications. Although pharmaceutical industry research focuses on green supply chain management (15) and digital transformation (16), it has failed to create associations between such micro-enterprise activities and macro-level goals for public health.

Third, the potential differential impacts of essential drug enterprises under the scope of green finance policies and their health outcomes have not yet been incorporated into the existing frameworks of research. Being the producers of products of public welfare, essential drug enterprises should receive the highest level of financial support in the policy domain, although this hypothesis is yet to be verified. More importantly, despite the potential improved financial conditions of essential drug enterprises, the key public health transmission mechanism of whether cost savings can transmit to essential drug prices and thereby benefit low-income groups has not been explored.

This study aims to fill the above gaps by using data from Chinese A-share listed drug manufacturing companies from 2016 to 2023. By using difference-in-differences models over multiple periods and chain mediation analysis, it examines how digital transformation affects the financing cost of drug companies through the mediated process of green certification and green finance. The analysis also focuses on its heterogeneous effect on essential drug companies and its implications for drug availability.

Research contributions are reflected at three levels:

In terms of theoretical contributions, this study integrates information asymmetry theory, signaling theory, and stakeholder theory to establish a theoretical framework regarding digital green finance empowerment's impact on financing costs. The information asymmetry theory argues that the application of information technology makes the achievement of green certification easier because of the reduction in the cost of gathering and confirming environmental data (18–20), signaling theory elucidates how green certification as a credible signal reduces adverse selection problems in financial markets (21, 22), and stakeholder theory identifies the role of various stakeholders in the dynamics of demand and supply of green finance (23–25). This study incorporates technology innovation, environmental governance, and financial assistance in one framework, and this increases the scope of research in green finance at the micro-level.

In terms of empirical contributions, this study examines for the first time from a health economics perspective the impact of green finance policies on pharmaceutical enterprises' financing costs and drug accessibility, revealing the differentiated effects of essential drug enterprises in policy benefits. It employs Bootstrap methods to test the chain mediation path of “digital transformation → green certification → green finance → financing costs,” quantifying the transmission mechanisms and relative contributions of each link. Focusing on the heterogeneous effects of essential drug enterprises, it uses instrumental variable methods and propensity score matching-difference-in-differences approaches to control endogeneity, enhancing the reliability of conclusions.

In terms of policy contributions, this study provides a financial innovation pathway for promoting the green transformation of the pharmaceutical industry, lowering the prices of drugs, and improving the accessibility of drugs, in order to promote the attainment of the “Healthy China” strategy. The study provides empirical evidence to improve the green finance regulatory guidelines and develop targeted policies to support the pharmaceutical industry. It demonstrates that green finance policies are capable of achieving environmental targets and indirectly contributing to health targets by decreasing costs of doing business. This corresponds to China's experience in realizing United Nations Sustainable Development Goal 3 (ensure healthy lives and promote wellbeing for all at all ages). The green finance mechanisms help to ensure price stability and drug availability in order to have policy tools to address health crises.

## 2 Theoretical framework and hypotheses

### 2.1 Theoretical foundation

Information asymmetry between pharmaceutical companies and financial institutions results in inefficient capital allocation (18). It is difficult to monitor environmental performance, and pharmaceutical industries have high monitoring costs (19). By using Internet of Things sensors, establishing a real-time monitoring network, and applying blockchain technologies to construct tamper-proof information tracing chain, it is possible for enterprises to prove the authenticity of their environmental pledge information at a lower cost, thus providing a favorable environment for enterprises to gain green certification (20). The theory of signaling provides a framework of analysis in which green certification affects corporate financing decisions. Successful signaling requires cost differentiation between high-quality and low-quality firms (21). Green certification creates high barriers for “greenwashing” enterprises (22), and reputation effects reinforce long-term credibility through continuous public supervision. Green finance is affected by various parties, including financial institutions that incorporate ESG criteria into their credit decisions (23); green investors who give more priority to sustainable projects and invest in them (24); and regulators use differentiated risk weights and fiscal incentives to form “positive incentives + negative constraints” (25).

### 2.2 Hypotheses development

The digital transformation makes it more possible for the pharmaceutical companies to achieve green certification because of the reduced marginal cost of collecting and providing information on the environment. The use of Internet of Things sensors makes it easy for the continuous monitoring of important environmental factors such as wastewater, waste gas, and energy consumption, greatly improving the availability and reliability of environmental data (26). The digital management system is able to provide relevant materials in a standardized and traceable form, thus reducing the workload during on-site audits for certification bodies and speeding up the certification process. Using blockchain technology can improve the resistance to tampering with data, providing a technical guarantee for the authenticity of the results of certification. The application of artificial intelligence algorithms in the area of environment risk warnings and energy consumption optimization reflects the capabilities of the firms in green management (27). Based on the above analysis, this study proposes H1: Digital transformation significantly increases pharmaceutical enterprises' probability of obtaining green certification.

Green certification is the conversion of business environmental performance into specific quality indicators through standardized assessment methods, which lowers the costs associated with screening in financial institutions (10). Policy-oriented financial institutions generally consider green certification a high-priority criterion for green credits. Commercial banks focus on environmental risk management in lending portfolios under more stringent environmental regulations, where environmentally certified firms are preferred by commercial banks due to their low environmental compliance risk. Investor sensitivity to certification information is higher in the green bonds market. Bond issuers holding authoritative green certifications can increase market acceptability of the green bonds, which may lead to decreased costs of issuance and underwriting fees (11). Based on the above analysis, this study proposes H2: Green certification significantly promotes pharmaceutical enterprises' acquisition of green finance support.

The reduction of financing costs through green finance is achieved through three mechanisms: policy-oriented rate preferences, risk premium reduction, and investor structure optimization. The People's Bank of China instructs commercial banks to provide financial support for green projects at a lower market rate in terms of policy instruments such as green refinancing special quotas and lower green credit risk-weighted standards (28). The local government fiscal interest subsidies and the guarantee credits of the green bonds' issuance relate to reducing the costs of financing bonds. Environmental transformation strategies such as environmental facilities and the use of clean production techniques reduce the risks of environmental fines and production stoppage by enterprises, as indicated by the enhanced credit ratings and increased distance to default (29). Green investors' preferences cause market clearing prices to be lower than ordinary bonds, forming a “green premium” phenomenon (12). Based on the above analysis, this study proposes H3: Green finance significantly reduces pharmaceutical enterprises' financing costs.

The impact of digital transformation on financing costs forms a chain mediation effect layer by layer along the complete chain of “digital transformation → green certification → green finance → financing costs”: digital technology applications reduce the information costs and time costs of obtaining green certification for enterprises, green certification provides the signal to receive qualifications and enjoys an advantage in the competitive market of green finance, the obtaining of green finance directly reduces the cost of finance (30). These three links complete a whole chain, and the interruption of any link weakens the final impact of digital transformation on financing costs. The chain mediation effect emphasizes the sequential relationship and process of the chain, consistent with the institutional setting under which this study is conducted, where companies have to obtain green certification prior to receiving support for green finance, and digital transformation is a prerequisite to promote the acquisition of green certification. Based on the above analysis, this study proposes H4: Green certification and green finance play a chain mediation role in the process of digital transformation affecting financing costs.

Essential drug enterprises may show greater policy effects in the above mechanisms. The reason for such heterogeneity is a great match between the attributes of priority in public health and policy-oriented finance in terms of goal orientation. The stability of essential drug enterprises has a direct relation to public health security, and it has legitimacy in terms of priority support by the government in resource allocation (31). The financial institution takes into account the performance of firms on social responsibility while allocating green credits. Firms that produce essential drugs receive social responsibility premiums due to the public welfare nature of their products, reflected in financial institutions' relatively higher tolerance of their environmental risks. Green investors not only assess investment targets on the environment but also on societal aspects. Green transformation in essential drug enterprises has dual benefits of being environmentally sustainable as well as socially equitable. Hence, it may attract impact investment funds as well as ESG-themed funds. In terms of public health benefits, lowering the funding costs for essential drug enterprises may have a more straightforward effect on reducing the prices of essential drugs. This is because essential drugs are mostly bulk drugs with high elasticities of demand. Hence, it will have a positive effect on health equity goals as it will help low-income classes as well as grass-roots health institutions. Based on the above analysis, this study proposes H5: The effect of essential drug enterprises reducing financing costs through digital transformation is significantly stronger than that of non-essential drug enterprises.

**Figure 1:**
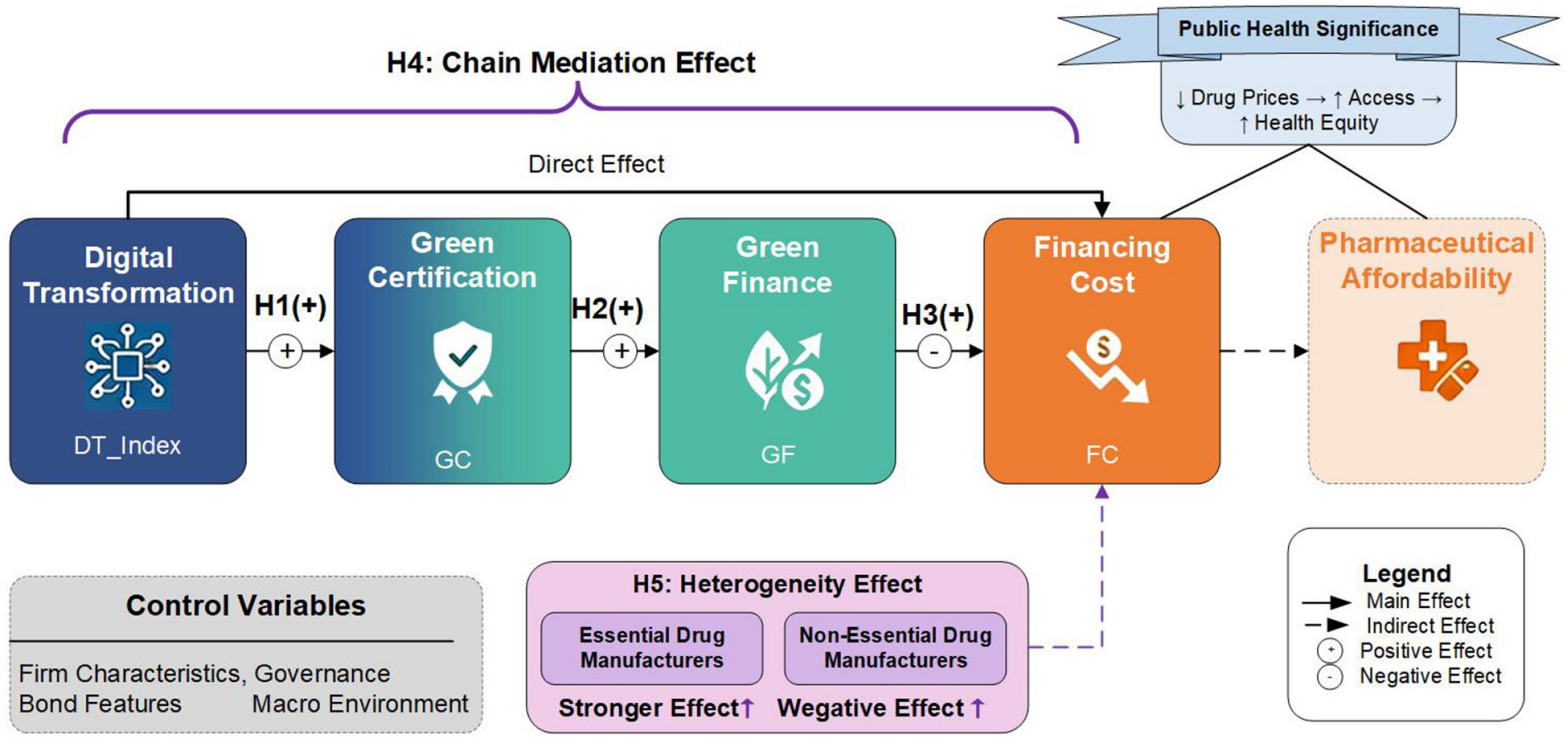
Conceptual framework of digital transformation, green finance, and pharmaceutical financing costs.

As shown in Figure 1, the conceptual framework established in this research presents the complete theoretical chain of the effect of digital transformation on the financing costs of pharmaceutical enterprises as it affects the accessibility of drugs via the chain of mediation of green certification and green finance. Digital transformation is the antecedent variable in this model as it assists enterprises in achieving green certification (path H1). Green certification as a signaling mechanism enhances enterprises' competitiveness in green finance markets (path H2). Green finance support reduces enterprise financing costs through policy preferences and market pricing mechanisms (path H3). The three constitute a complete chain mediation path (path H4). The framework also specifically marks the moderating effect of essential drug enterprises (path H5), which highlights the importance of this diverse effect in achieving health equity goals. The framework systematically combines micro-enterprise dynamics with macro-level health goals and provides a theoretical explanation of how green finance policies are related to enhanced drug availability.

## 3 Data and methodology

### 3.1 Sample selection

This study takes Chinese A-share listed pharmaceutical manufacturing enterprises from 2016–2023 as research subjects. The sample period starting point is selected based on the promulgation of the “Guiding Opinions on Establishing a Green Financial System” in 2016, which marked the entry of China's green finance policy into a systematic implementation stage, with the endpoint set at 2023 to ensure data completeness (32). According to the China Securities Regulatory Commission's 2012 industry classification standard, all listed companies with industry code C27 (pharmaceutical manufacturing) are selected as the initial sample. Sample screening retains bond-issuing firms, excludes ST/delisted firms and observations with >30% missing data (33). After screening, 1,216 firm-year observations from 152 pharmaceutical manufacturing listed companies are obtained, constituting a balanced panel dataset. It should be noted that the sample is restricted to firms that issued bonds during the study period, as the dependent variable (financing cost) is based on bond yield spreads. Bond-issuing firms tend to be larger, have higher leverage ratios, and are more likely to be state-owned compared with non-issuing pharmaceutical firms. These characteristics suggest that the sample represents relatively larger and more established pharmaceutical companies, which may limit the generalizability of findings to smaller firms and non-listed enterprises. The implications of this sample selection are further discussed in Section 5.4.

The sources of data used for this study include the CSMAR database and the Wind Information database. The CSMAR database offers financial statement information for listed corporations, corporate governance information, and basic bond issuance information, while the Wind offers bond market trading data, financing cost indicators, and macroeconomic variables. The digital transformation index can be obtained through text analysis method. The PDF files of annual reports of sample firms are extracted from Cninfo, and Python is used for text preprocessing and keyword identification. Based on the digital transformation keyword dictionary (see Appendix A for the complete list), which includes 32 keywords in two categories: underlying technology terms (such as artificial intelligence, big data, cloud computing, blockchain, and Internet of Things) and application terms (such as intelligent manufacturing, industrial internet, and digital management), the total keyword frequency is calculated for each firm-year, and the natural logarithm is taken after adding 1 to constitute the enterprise annual digital transformation index. The keyword dictionary follows the framework established in prior literature (34) with minor adaptations for the pharmaceutical industry context. Green certification information comes from the Ministry of Industry and Information Technology's green manufacturing list, the Ministry of Ecology and Environment's environmentally friendly enterprise certification database, and ISO 14001 environmental management system certification records, constructing enterprise annual dummy variables through manual collection and verification. Essential drug enterprise identification is based on the National Health Commission's “National Essential Medicines List.” A firm is classified as an essential drug enterprise if its main products, as disclosed in its annual report, include varieties listed in the National Essential Medicines List and these products account for a significant share of the firm's pharmaceutical business. The classification primarily follows the 2018 edition, which expanded the list from 520 to 685 varieties. For the sample years 2016–2017, prior to the 2018 revision, classification was based on the 2012 edition. Product-catalog matching was conducted at the generic drug name level, with manual verification for cases involving combination formulations or name variations.

### 3.2 Variable definitions

Financing cost equals bond coupon rate minus treasury yield of same maturity and rating, weighted by issuance scale (35). Digital transformation uses ln (1 + keyword frequency) from annual reports (34). Green certification is a binary indicator. Green finance (GF) is measured by a binary indicator of whether the firm issued green bonds in a given year, identified through the Wind database and verified manually against bond prospectuses. Green bond issuance is selected as the proxy for green finance for three reasons. First, green bonds have the most standardized identification criteria and transparent disclosure requirements among green finance instruments in China, enabling reliable data collection and verification (7). Second, green bond issuance represents a firm's formal entry into the green finance market and typically signals broader access to green financial resources, including preferential green credit terms. Third, alternative green finance data, particularly firm-level green loan information, is not systematically disclosed by Chinese listed companies, making comprehensive measurement infeasible. The limitations of this measure are discussed in Section 5.4.

Control variable selection follows the theoretical framework of enterprise financing cost determinants, including enterprise size (natural logarithm of total assets), profitability (ROA), asset-liability ratio, cash holding ratio (monetary funds divided by total assets), growth (operating revenue growth rate), enterprise age (natural logarithm of listing years), property rights nature dummy variable, board size, independent director ratio, provincial GDP growth rate, and national CPI (36). The selection of all control variables is justified by relevant literature and is able to distinguish the impact of various confounding variables on relationships between key variables (37). The grouping variable is a crucial dummy variable in the pharmaceutical industry and is used to examine differential policy impacts in heterogeneity analysis.

### 3.3 Econometric models

The benchmark regression model adopts a fixed-effects panel data model, with the following three equations set up to test hypotheses H1 through H3 respectively (Equations 1–3):


$$\begin{array}{l} GC_{it}=\alpha_{0}+\beta_{1}DT_{it}+\gamma X_{it}+\mu_{i}+\lambda_{t}+\varepsilon_{it} \end{array}$$ (1)

$$\begin{array}{l} GF_{it}=\alpha_{0}+\beta_{2}GC_{it}+\gamma X_{it}+\mu_{i}+\lambda_{t}+\varepsilon_{it} \end{array}$$ (2)

$$\begin{array}{l} FC_{it}=\alpha_{0}+\beta_{3}GF_{it}+\gamma X_{it}+\mu_{i}+\lambda_{t}+\varepsilon_{it} \end{array}$$ (3)

where *i* represents enterprises, *t* represents years, *GC*_*it*_, *GF*_*it*_, and *FC*_*it*_ represent green certification, green finance, and financing costs respectively, *DT*_*it*_ is the digital transformation index, *X*_*it*_ is the control variable vector, μ_*i*_ is the enterprise fixed effect used to control time-invariant enterprise heterogeneity, λ_*t*_ is the year fixed effect used to control macroeconomic shocks and time trends in policy environment, and ε_*it*_ is the random error term (38). The standard errors are corrected for clustering on the enterprise level to address possible serial correlation and heteroscedasticity of observations of the same enterprise for different years. The chain mediation model assumes a sequential ordering of digital transformation, green certification, and green finance. This temporal assumption is supported by China's institutional context. Under current regulations, firms must first obtain third-party green certification before applying for green bond issuance, as certification serves as a prerequisite in the green bond verification process required by the People's Bank of China and the National Development and Reform Commission (7, 28). Digital transformation, as a technology adoption process, logically precedes environmental data collection and certification application. While the annual data structure cannot establish precise within-year ordering, the institutional sequence provides a reasonable basis for the assumed causal direction. As a robustness check, we also tested models using 1 year lagged independent variables. The coefficient of lagged digital transformation on green certification is 0.071 (*p* < 0.01), and the coefficient of lagged green certification on green finance is 0.118 (*p* < 0.01), both qualitatively consistent with the baseline results.

The test of chain mediation effects uses the Bootstrap method, in which an empirical distribution of mediation effects is derived from resampling. This method avoids the problem of requiring normality in the Sobel test and has a larger statistical power in finite samples (39). The complete mediation effect model is specified as:


$$\begin{array}{l} FC_{it}=c_{0}+c_{1}DT_{it}+\gamma X_{it}+\mu_{i}+\lambda_{t}+\varepsilon_{it} \end{array}$$ (4)

$$\begin{array}{l} GC_{it}=a_{1}+a_{2}DT_{it}+\gamma X_{it}+\mu_{i}+\lambda_{t}+\varepsilon_{it} \end{array}$$ (5)

$$\begin{array}{l} GF_{it}=b_{1}+b_{2}GC_{it}+\gamma X_{it}+\mu_{i}+\lambda_{t}+\varepsilon_{it} \end{array}$$ (6)

$$\begin{array}{l} FC_{it}=d_{1}+d_{2}GF_{it}+\gamma X_{it}+\mu_{i}+\lambda_{t}+\varepsilon_{it} \end{array}$$ (7)

$$\begin{array}{l} FC_{it}=e_{1}+e_{2}DT_{it}+e_{3}GC_{it}+e_{4}GF_{it}+\gamma X_{it}+\mu_{i}+\lambda_{t}+\varepsilon_{it} \end{array}$$ (8)

Equation 4 estimates the total effect *c*_1_ of digital transformation on financing costs. Equation 5 estimates the effect of digital transformation on green certification, Equation 6 estimates the effect of green certification on green finance, and Equation 7 estimates the effect of green finance on financing costs. Equation 8 incorporates all variables simultaneously to test the completeness of the mediation effect. The existence condition for chain mediation effect is that *a*_2_, *b*_2_, and *d*_2_ are all significant and the Bootstrap confidence interval of the product *a*_2_×*b*_2_×*d*_2_ does not include zero (40). Bootstrap sampling is set at 5,000 repetitions with a confidence level of 95% to ensure the reliability of statistical inference.

Heterogeneity analysis tests the moderating effect of essential drug enterprises by introducing interaction terms, with the model specified as follows (Equation 9):


$$\begin{array}{l} FC_{it}=\alpha_{0}+\beta_{1}DT_{it}+\beta_{2}EMM_{i}+\beta_{3}\left(DT_{it}\times EMM_{i}\right)+\\ \;\gamma X_{it}+\mu_{i}+\lambda_{t}+\varepsilon_{it} \end{array}$$ (9)

where *EMM*_*i*_ is the essential drug enterprise dummy variable, and the interaction term coefficient β_3_ measures the marginal difference in the effect of digital transformation on reducing financing costs for essential drug enterprises compared to non-essential drug enterprises. If β_3_ is significantly negative, it indicates that the effect of essential drug enterprises in reducing the cost of financing through digital transformation is more significant, hence supporting H5.

Endogeneity treatment adopts the instrumental variable method and propensity score matching-difference-in-differences (PSM-DID). The instrumental variables method employs the local internet penetration rate for the city where the company is located as an instrument for digital transformation and uses two-stage least squares (2SLS) to correct for endogeneity issues associated with digital transformation. The relevance condition is satisfied because regional internet infrastructure facilitates firm-level adoption of digital technologies such as cloud computing, big data analytics, and IoT systems, as confirmed by the first-stage F-statistic of 34.67, well above the conventional threshold of 10 for weak instruments (38). Regarding the exclusion restriction, we argue that city-level internet penetration affects pharmaceutical firms‘ bond financing costs primarily through firm-level digital transformation rather than through direct channels, for the following reasons. First, the baseline model already controls for provincial GDP growth rate, which captures the general level of regional economic development that might correlate with both internet infrastructure and financing conditions. Second, internet penetration reflects telecommunications infrastructure that is largely determined by historical policy decisions regarding network deployment, rather than by contemporaneous financial market conditions. Third, pharmaceutical firms' bond pricing is primarily determined by firm-specific financial characteristics and industry factors rather than by local internet availability. The Sargan test (*p* = 0.387) provides additional statistical support for the validity of the instrument. To further address potential concerns about the identification strategy, this study employs multiple complementary approaches. In addition to the IV-2SLS method, the PSM-DID approach provides an alternative identification framework by exploiting the quasi-exogenous variation in the timing of green certification acquisition. The PSM-DID method models the process of obtaining green certification as a quasi-natural experiment. In the matching stage, treated firms (those obtaining green certification) are matched with control firms using 1:1 nearest-neighbor matching without replacement, based on propensity scores estimated from pre-treatment firm characteristics including firm size, profitability, leverage, cash holdings, growth rate, firm age, ownership type, and digital transformation level. The matching effectively reduces the mean standardized bias from 12.34% to 3.67% across covariates, indicating satisfactory balance between treated and control groups. In the estimation stage, the treatment effect is identified through a difference-in-differences model applied to the matched sample, exploiting the staggered timing of green certification across firms (40). The parallel trends assumption is verified through dynamic DID estimates (reported in Section 4.4), where pre-treatment coefficients (t-3 to t-1) are statistically insignificant.

Robustness checks include alternative dependent variable (interest expense ratio), subsample exclusions (SOEs, large firms, high-leverage firms), and alternative winsorization (2%/98%). Mechanism tests employ analyst coverage and stock illiquidity to proxy information asymmetry, credit rating upgrades and Altman Z-score to proxy risk premium. Parallel trends are tested via dynamic DID; placebo tests randomize certification timing (1,000 simulations).

### 3.4 Descriptive statistics

The basic characteristics of the sample firms are shown in Table 1. The average financing cost is 2.847% with a standard deviation of 1.523%, which shows great variability in the financing costs of the pharmaceutical manufacturing listed firms in the bond market. The minimum and maximum financing cost is 12 and 785 basis points, respectively. This heterogeneity provides an appropriate variance context under which the impact of digital transformation and green certification on financing costs can be studied. The average value of the digital transformation index is 2.134 with a standard deviation of 1.287, which reveals large disparities regarding the intensity of disclosures of digital technology applications by pharmaceutical companies. Approximately 30.9% of the enterprises have obtained green certification, and 14.7% of them have issued green bonds. The results show that the pharmaceutical industry is still in a developmental stage regarding green and digital upgrading. The enterprises engaged in essential drugs account for 44.7% of the sample and are balanced samples for testing heterogeneity. The distributional characteristics of control variables are consistent with the usual features found in the pharmaceutical industry. The mean value of the asset-liability ratio of 38.5% is within a reasonable zone, and the mean ROA of 5.6% is higher than that of the manufacturing industry, signifying that the pharmaceutical industry has relatively high profitability.

**Table 1:** Variable definitions and descriptive statistics.

Variable	Definition	Measurement	Mean	Std. Dev.	Min	Max
Dependent variable						
FC	Financing Cost	Bond yield spread (%)	2.847	1.523	0.120	7.850
Independent variables						
DT	Digital Transformation	ln(1 + keyword frequency)	2.134	1.287	0.000	5.621
GC	Green Certification	1 if certified, 0 otherwise	0.309	0.462	0	1
Mediating variable						
GF	Green Finance	1 if issued green bonds, 0 otherwise	0.147	0.354	0	1
Moderating variable						
EMM	Essential Medicine	1 if essential drug manufacturer, 0 otherwise	0.447	0.497	0	1
Control variables						
Size	Firm size	ln(Total assets)	22.456	1.234	19.678	26.342
ROA	Profitability	Net income/Total assets (%)	5.623	6.789	−15.234	24.567
Lev	Leverage	Total liabilities/Total assets (%)	38.456	18.234	5.678	82.345
Cash	Cash holdings	Cash/Total assets (%)	18.234	12.456	2.345	56.789
Growth	Sales growth	Revenue growth rate (%)	12.345	23.456	−45.678	98.765
Age	Firm age	ln(Years since IPO)	2.234	0.567	0.693	3.456
SOE	State Ownership	1 if state-owned, 0 otherwise	0.428	0.495	0	1
Board	Board size	Number of directors	8.567	1.789	5	15
Indep	Independent directors	Proportion of independent directors (%)	37.234	5.123	33.333	60.000
GDP	GDP growth	Provincial GDP growth rate (%)	6.234	1.456	2.345	10.234
CPI	Inflation	Consumer price index (%)	102.345	1.789	99.234	106.789

*N* = 1,216 firm-year observations from 152 pharmaceutical manufacturing firms, 2016–2023.

The grouped comparison results are shown in Table 2. Panel A shows the correlation coefficients for the key variables. The digital transformation index, green certification, and green finance show significant negative correlations with financing costs, thus initially verifying the hypotheses for this study. However, the absolute values for the correlation coefficients are all less than 0.5, thus indicating a lack of serious multicollinearity for the variables (41). The correlation coefficient for digital transformation and green certification is 0.456, and it is 0.378 for digital transformation and green finance. This shows that there is a positive correlation between digital transformation and the acquisition of green certification and support for green finance. This is evidence for the potential mediation roles. On the correlation of control variables, it can be seen that size and profit have negative correlations with costs of finance, while asset-liability has a positive correlation, which is expected.

Table 2Correlation matrix and group comparisons.

**Table:** 

Panel A: correlation matrix								
	(1)	(2)	(3)	(4)	(5)	(6)	(7)	(8)
(1) FC	1.000							
(2) DT	−0.234^***^	1.000						
(3) GC	−0.312^***^	0.456^***^	1.000					
(4) GF	−0.287^***^	0.378^***^	0.523^***^	1.000				
(5) Size	−0.198^***^	0.287^***^	0.234^***^	0.312^***^	1.000			
(6) ROA	−0.345^***^	0.156^***^	0.178^***^	0.145^***^	0.234^***^	1.000		
(7) Lev	0.456^***^	−0.123^***^	−0.156^***^	−0.134^***^	0.234^***^	−0.456^***^	1.000	
(8) Growth	−0.089^**^	0.145^***^	0.098^**^	0.087^**^	−0.034	0.278^***^	−0.145^***^	1.000

**Table:** 

Panel B: group comparisons (essential medicine vs. non-essential medicine manufacturers)				
Variable	Essential medicine (*N* = 543)	Non-essential medicine (*N* = 673)	Difference	t-statistic
FC	2.634	3.012	−0.378^***^	−3.456
DT	2.287	2.012	0.275^***^	2.987
GC	0.356	0.271	0.085^***^	2.567
GF	0.178	0.123	0.055^**^	2.234
Size	22.567	22.367	0.200^*^	1.876
ROA	5.987	5.334	0.653^**^	2.345
Lev	36.234	40.123	−3.889^***^	−2.987

^***^, ^**^, ^*^ denote significance at 1%, 5%, and 10% levels, respectively.

Panel B presents the mean difference test results of key variables between essential drug enterprises and non-essential drug enterprises. The average financing cost for essential drug enterprises is found to be significantly lower than that for non-essential drug enterprises by about 38 basis points (t = −3.456, *p* < 0.01). At the same time, essential drug enterprises were found to have higher levels in the digital transformation index, the percentage of green certification, and the degree of green finance. This difference might be due to the public health attributes of essential drug enterprises, which give them priority in policy-oriented financial support, or it might be due to higher motivations and abilities in green transformation and digital upgrading of such enterprises. The average asset-liability ratio of essential drug enterprises is much lower than that of non-essential drug enterprises by about 3.9 percentage points (t = −2.987, *p* < 0.01), which indicates relatively low risk in finance. However, this variable will be considered and filtered by using control variables in multivariate analysis.

## 4 Results

### 4.1 Baseline regressions

Table 3 presents baseline results. Digital transformation significantly increases green certification probability (β = 0.079, t = 3.87, *p* < 0.01), validating H1. Green certification promotes green finance (β = 0.134, t = 5.67, *p* < 0.01), validating H2. Green finance reduces financing costs by 48.7 bp (β = −0.487, t = −6.34, *p* < 0.01), validating H3. Based on the average interest-bearing debt of ¥1.5 billion for sample firms (calculated from CSMAR data), the 48.7 bp reduction translates to approximately ¥7.3M annual savings for average firms, which could potentially create price adjustment room, particularly for essential drug enterprises operating under government price regulation.

**Table 3:** Baseline regression results for hypotheses H1–H3.

Variable	H1: DT→GC			H2: GC→GF			H3: GF→FC		
	(1)	(2)	(3)	(4)	(5)	(6)	(7)	(8)	(9)
DT	0.087^***^ (4.23)	0.082^***^ (3.98)	0.079^***^ (3.87)						
GC				0.142^***^ (5.89)	0.137^***^ (5.73)	0.134^***^ (5.67)			
GF							−0.502^***^ (−6.78)	−0.493^***^ (−6.51)	−0.487^***^ (−6.34)
Size		0.023^**^ (2.11)	0.021^*^ (1.89)		0.058^***^ (3.34)	0.056^***^ (3.21)		−0.241^***^ (−4.67)	−0.234^***^ (−4.56)
ROA		0.003^**^ (2.34)	0.003^**^ (2.28)		0.002 (1.45)	0.002 (1.38)		−0.041^***^ (−4.01)	−0.038^***^ (−3.89)
Lev		−0.001^*^ (−1.87)	−0.001^*^ (−1.79)		−0.002^**^ (−2.41)	−0.002^**^ (−2.34)		0.026^***^ (5.89)	0.024^***^ (5.67)
Cash		0.001 (1.23)	0.001 (1.19)		0.001 (0.87)	0.001 (0.83)		−0.008^**^ (−2.11)	−0.007^**^ (−2.03)
Growth		0.000 (0.45)	0.000 (0.42)		0.000 (0.56)	0.000 (0.54)		−0.003^*^ (−1.78)	−0.003^*^ (−1.72)
Age		−0.012 (−1.34)	−0.011 (−1.28)		−0.018 (−1.23)	−0.017 (−1.19)		0.087^**^ (2.34)	0.083^**^ (2.26)
SOE		0.034^**^ (2.11)	0.033^**^ (2.05)		0.028^*^ (1.78)	0.027^*^ (1.72)		−0.156^***^ (−3.21)	−0.149^***^ (−3.12)
Board			0.005 (1.12)			0.004 (0.98)			−0.034^*^ (−1.89)
Indep			0.002 (0.87)			0.002 (0.79)			−0.015 (−1.43)
GDP			0.006 (1.34)			0.005 (1.21)			−0.045^*^ (−1.78)
CPI			−0.003 (−0.56)			−0.002 (−0.48)			0.023 (1.12)
Firm FE	Yes	Yes	Yes	Yes	Yes	Yes	Yes	Yes	Yes
Year FE	Yes	Yes	Yes	Yes	Yes	Yes	Yes	Yes	Yes
N	1,216	1,216	1,216	1,216	1,216	1,216	1,216	1,216	1,216
R^2^	0.267	0.289	0.293	0.312	0.334	0.338	0.423	0.467	0.471
F-statistic	12.34^***^	11.89^***^	10.67^***^	15.67^***^	14.23^***^	13.45^***^	18.90^***^	17.34^***^	16.23^***^

t-statistics in parentheses. ^***^, ^**^, ^*^ denote significance at 1%, 5%, and 10% levels, respectively. Standard errors clustered at firm level.

### 4.2 Mediation analysis

Table 4 presents mediation analysis using Bootstrap method (5,000 replications). Total effect: −0.038 (95% CI: [−0.052, −0.024], *p* < 0.01). Direct effect: −0.011 (95% CI: [−0.026, 0.004], n.s.). Total indirect effect: −0.027 (95% CI: [−0.038, −0.016], *p* < 0.01), accounting for 71.1% of total effect, confirming mediation dominates the mechanism. Three pathways contribute: Path 1 (DT → GC → FC): −0.006 (95% CI: [−0.011, −0.002]), 15.8% of total. Path 2 (DT → GF → FC): −0.008 (95% CI: [−0.014, −0.003]), 21.1% of total. Path 3 chain mediation (DT → GC → GF → FC): −0.013 (95% CI: [−0.021, −0.006], *p* < 0.01), 34.2% of total, thus validating H4.

**Table 4:** Mediation analysis results: bootstrap test of indirect effects.

Effect type	Coefficient	Std. error	95% CI lower	95% CI upper	Proportion of total effect
Total effect	−0.038^***^	0.007	−0.052	−0.024	100.0%
Direct effect	−0.011	0.008	−0.026	0.004	28.9%
Total indirect effect	−0.027^***^	0.006	−0.038	−0.016	71.1%
Specific indirect effects:					
Path 1: DT → GC → FC	−0.006^**^	0.002	−0.011	−0.002	15.8%
Path 2: DT → GF → FC	−0.008^**^	0.003	−0.014	−0.003	21.1%
Path 3: DT → GC → GF → FC	−0.013^***^	0.004	−0.021	−0.006	34.2%

Bootstrap standard errors and 95% confidence intervals based on 5,000 replications. ^***^, ^**^ denote significance at 1% and 5% levels when confidence intervals exclude zero. DT, Digital Transformation; GC, Green Certification; GF, Green Finance; FC, Financing Cost.

### 4.3 Heterogeneity analysis

Table 5 presents heterogeneity analysis. Essential drug manufacturers show significantly stronger effects (β = −0.051, t = −4.12) than non-essential manufacturers (β = −0.027, t = −1.89), approximately 1.9 times larger (Chow F = 3.67, *p* < 0.05), validating H5. This result has several potential implications for public health concerning drug availability, as discussed in Section 5. Panels B–D show no significant heterogeneity across ownership, size, or regions.

**Table 5:** Heterogeneity analysis by firm characteristics and regions.

Variable	Panel A: essential drug status		Panel B: ownership		Panel C: firm size		Panel D: region	
	Essential	Non-essential	SOE	Non-SOE	Large	Small	East	Central-West
DT	−0.051^***^ (−4.12)	−0.027^*^ (−1.89)	−0.034^***^ (−2.78)	−0.042^***^ (−3.21)	−0.045^***^ (−3.56)	−0.029^**^ (−2.11)	−0.043^***^ (−3.67)	−0.031^**^ (−2.34)
Controls	Yes	Yes	Yes	Yes	Yes	Yes	Yes	Yes
Firm FE	Yes	Yes	Yes	Yes	Yes	Yes	Yes	Yes
Year FE	Yes	Yes	Yes	Yes	Yes	Yes	Yes	Yes
N	543	673	520	696	608	608	789	427
R^2^	0.487	0.452	0.468	0.473	0.491	0.441	0.479	0.456
Chow test F	3.67^**^		0.67		1.89		1.45	
Chow test p	0.026		0.412		0.169		0.229	

t-statistics in parentheses. ^***^, ^**^, ^*^ denote significance at 1%, 5%, and 10% levels. All specifications include full controls as in Table 3 Column (9).

**Figure 2:**
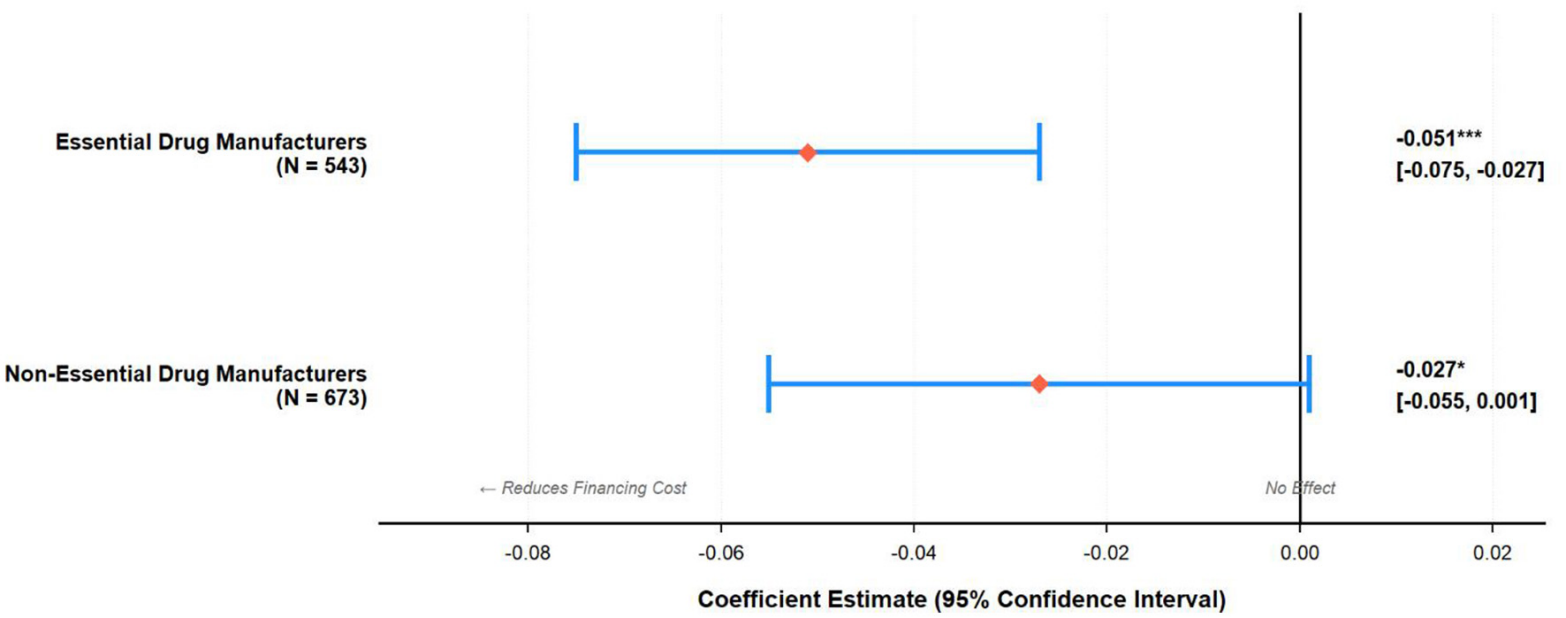
Heterogeneous effects of digital transformation on financing costs by essential drug manufacturer status: coefficient estimates and 95% confidence intervals.

Figure 2 forest plot shows non-overlapping 95% confidence intervals between essential and non-essential drug firms, hence establishing significant heterogeneity consistent with the findings for the interaction terms (β = −0.024, t = −2.18, *p* < 0.05).

### 4.4 Robustness and endogeneity tests

Table 6 presents endogeneity tests. Panel A: IV-2SLS using city internet penetration (first-stage F = 34.67) yields second-stage coefficient −0.046 (t = −2.34, *p* < 0.05). Sargan test (*p* = 0.387) supports exogeneity. Panel B: PSM-DID with 1:1 matching reduces standardized bias from 12.34% to 3.67%. DID coefficient −0.412 (t = −4.23, *p* < 0.01) confirms baseline results.

**Table 6:** Endogeneity tests: instrumental variable and PSM-DID.

Variable	Panel A: IV-2SLS		Panel B: PSM-DID	
	First stage	Second stage	Before match	After match
	DT	FC	FC	FC
Internet penetration	0.127^***^ (5.89)			
DT (Instrumented)		−0.046^**^ (−2.34)		
GC × Post			−0.387^***^ (−3.89)	−0.412^***^ (−4.23)
Controls	Yes	Yes	Yes	Yes
Firm FE	Yes	Yes	Yes	Yes
Year FE	Yes	Yes	Yes	Yes
N	1,216	1,216	1,216	618
R^2^/Pseudo R^2^	0.512	0.468	0.471	0.489
First-stage F	34.67^***^			
Sargan test p		0.387		
Mean Std. Bias			12.34%	3.67%

t-statistics in parentheses. ^***^, ^**^ denote significance at 1% and 5% levels. IV, Instrumental Variable; PSM-DID, Propensity Score Matching Difference-in-Differences.

**Figure 3:**
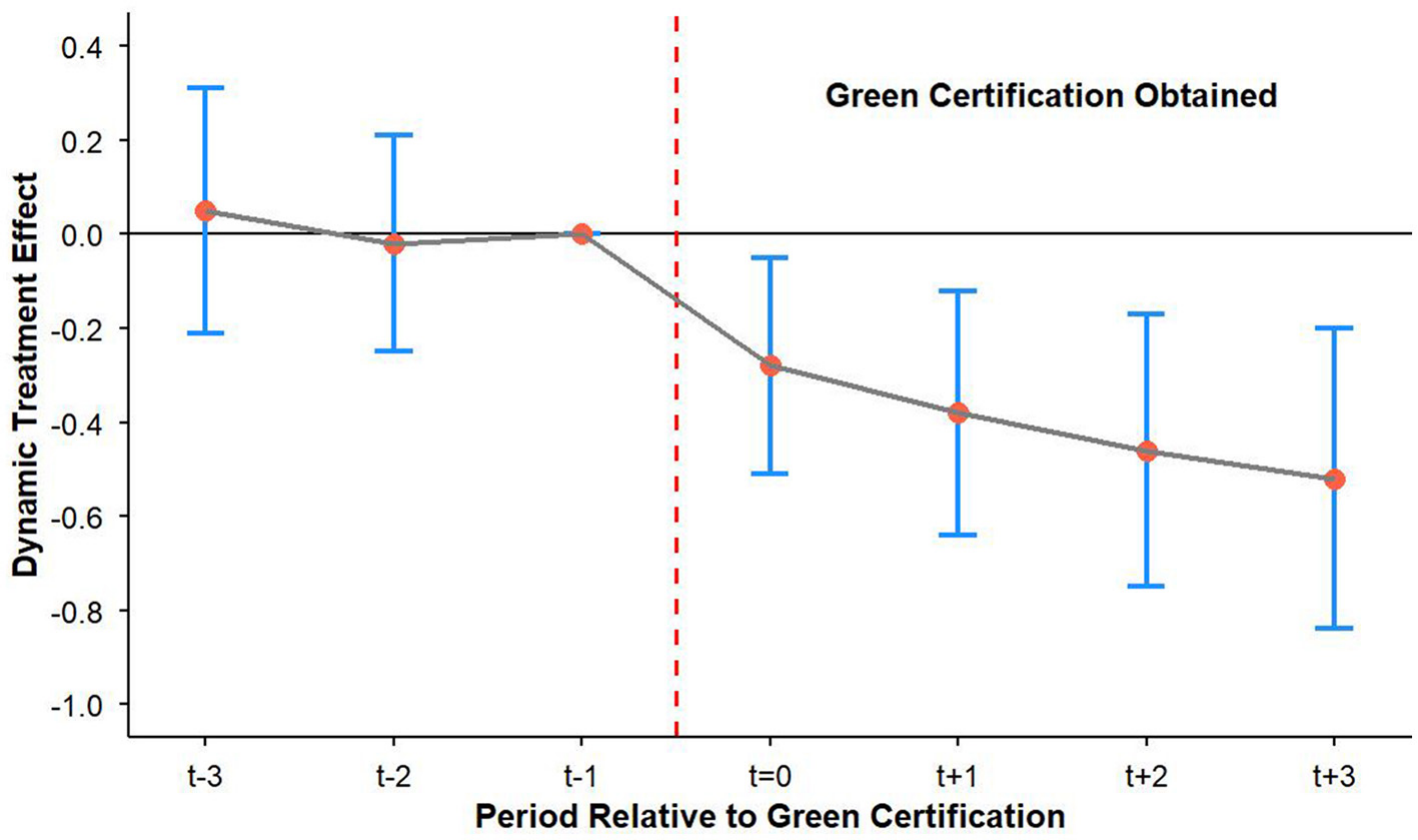
Parallel trend test for green certification treatment: dynamic difference-in-differences estimates with 95% confidence intervals.

Figure 3 dynamic DID shows parallel pre-trends (t = −3 to t = −1 coefficients insignificant) and significant post-treatment effects (t = 0 to t = 3), confirming causal identification.

Table 7 validates robustness through: (1) alternative DV (interest expense ratio), (2–4) excluding SOEs/large firms/high-leverage firms, (5) alternative winsorization. All specifications show significant negative DT coefficients.

**Table 7:** Robustness checks: alternative specifications and samples.

Variable	(1)	(2)	(3)	(4)	(5)	(6)
	Baseline	Alt. DV	Excl. SOE	Excl. Large	Excl. High Lev	Alt. Winsor
DT	−0.038^***^(−3.45)	−0.003^**^(−2.21)	−0.042^***^(−3.21)	−0.035^**^(−2.67)	−0.041^***^(−3.45)	−0.036^***^(−3.12)
Controls	Yes	Yes	Yes	Yes	Yes	Yes
Firm FE	Yes	Yes	Yes	Yes	Yes	Yes
Year FE	Yes	Yes	Yes	Yes	Yes	Yes
N	1,216	1,216	696	912	1,089	1,216
R^2^	0.471	0.423	0.485	0.462	0.478	0.469

t-statistics in parentheses. ^***^, ^**^ denote significance at 1% and 5% levels. Alt. DV, Alternative dependent variable (interest expense ratio); Excl., Excluding; Alt. Winsor, Alternative winsorization at 2% and 98%.

**Figure 4:**
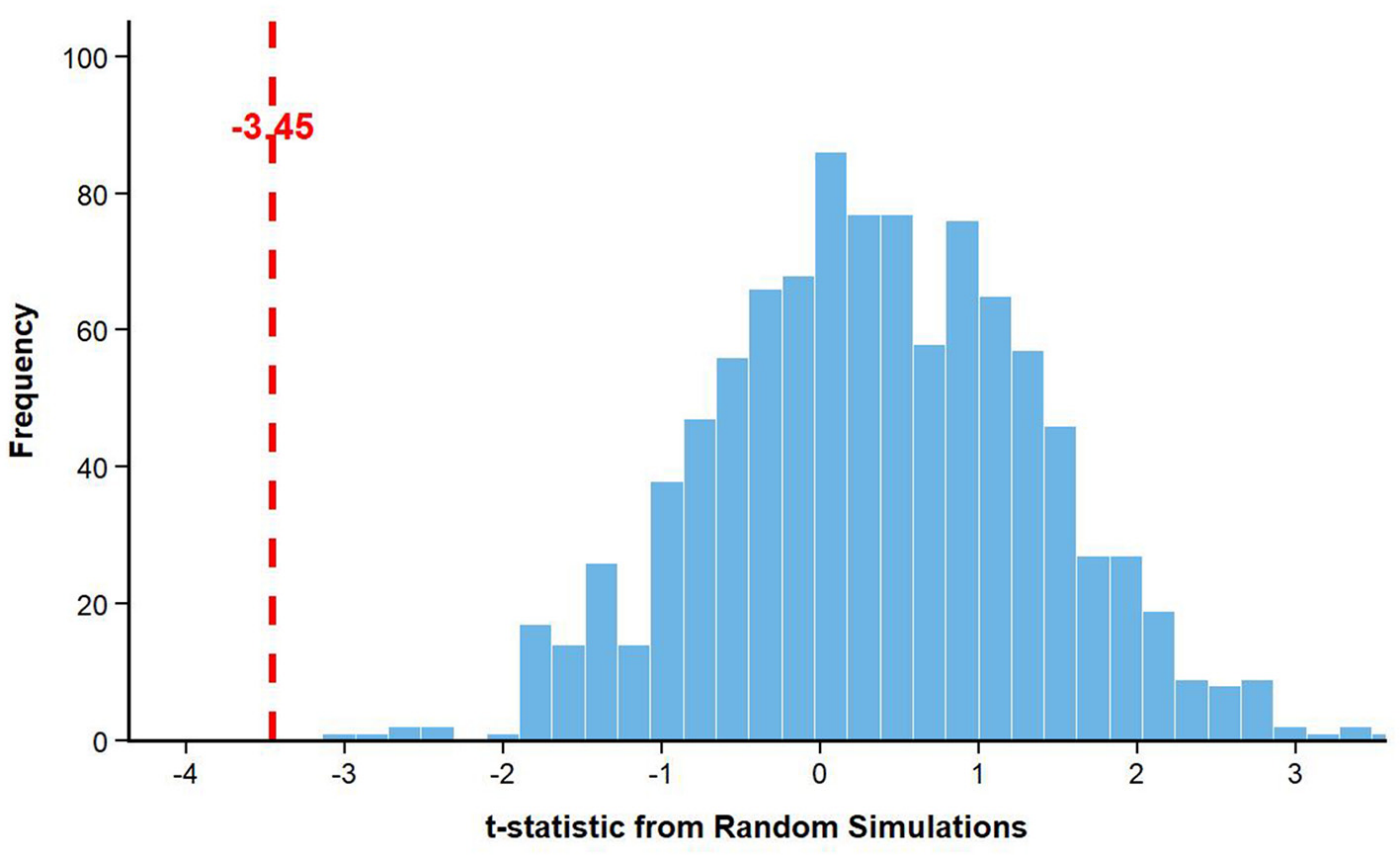
Placebo test distribution: t-statistics from 1,000 random simulations vs. actual baseline estimate.

Figure 4 placebo test randomizes green certification timing across 1,000 simulations. Actual t-statistic (−3.45) falls in extreme tail (*p* < 0.05), rejecting random chance.

### 4.5 Mechanism tests

Mechanism tests examine how digital transformation affects financing costs through micro-channels by employing proxy variables that reflect information asymmetry and risk premium. Theoretically, there are two possible action channels that can be distinguished: information asymmetry mitigation mechanism and risk premium reduction mechanism. As shown in Table 8, Panel A examines the information asymmetry mitigation mechanism. The coefficient of digital transformation on analyst followers is 0.124 (t = 3.67, *p* < 0.01). The coefficient on stock illiquidity is −0.087 (t = −2.89, *p* < 0.01). Panel B tests the risk premium reduction mechanism. The coefficient of digital transformation on credit rating upgrades is 0.091 (t = 2.78, *p* < 0.01). The coefficient on distance to default is 0.156 (t = 3.45, *p* < 0.01). The proxy variables of information asymmetry and risk premium are employed as the mediating variables in the cost of financing equation in Panel C. The direct effect of these two channels on the cost of financing is examined. The information asymmetry channel explains around 39% of the total effect. The risk premium effect is approximately 53% of the total effect. Cumulatively, the two channels explain approximately 92% of the total effect, indicating that the mitigation of information asymmetry and the reduction of the risk premium are the dominant micro-mechanisms through which the cost of financing is reduced by the digital transformation.

**Table 8:** Mechanism tests: information asymmetry and risk premium channels.

Variable	Panel A: Information asymmetry		Panel B: Risk premium		Panel C: Financing cost	
	(1)	(2)	(3)	(4)	(5)	(6)
	Analyst coverage	Illiquidity	Rating upgrade	Distance to default	FC	FC
DT	0.124^***^ (3.67)	−0.087^***^ (−2.89)	0.091^***^ (2.78)	0.156^***^ (3.45)	−0.023 (−1.45)	−0.018 (−1.21)
Analyst coverage					−0.067^**^ (−2.34)	
Distance to default						−0.089^***^ (−3.12)
Controls	Yes	Yes	Yes	Yes	Yes	Yes
Firm FE	Yes	Yes	Yes	Yes	Yes	Yes
Year FE	Yes	Yes	Yes	Yes	Yes	Yes
N	1,216	1,216	1,216	1,216	1,216	1,216
R^2^	0.512	0.478	0.423	0.534	0.483	0.487

t-statistics in parentheses. ^***^, ^**^ denote significance at 1% and 5% levels. Analyst Coverage = ln(1 + number of analysts); Illiquidity, Amihud illiquidity measure; Rating Upgrade, 1 if credit rating upgraded, 0 otherwise; Distance to Default, Altman Z-score.

### 4.6 Potential public health implications

On the basis of the above empirical evidence, the digital transformation reduces the cost of financing substantially for the pharmaceutical manufacturing enterprises in the industry through the encouragement of green certification and the promotion of the availability of green finance. This is achieved through two micro-mechanism channels, namely the reduction of information asymmetry and the reduction of risk premium, and is more evident in the enterprises manufacturing essential drugs. The cost reduction may provide room for the adjustment of prices. Under China's essential drug price regulation framework, the reduction in financing costs for essential drug enterprises could theoretically be more readily transmitted to final drug prices due to the product's welfare attributes and government pricing oversight. If realized, such price reductions for essential medications would be beneficial to low-income people and grassroots medical institutions, potentially contributing to progress toward health equity goals. However, the actual transmission from financing cost reduction to drug price adjustment was not directly tested in this study and remains an important area for future empirical investigation. This finding provides evidence of the role of green finance policies in reducing pharmaceutical enterprises' financing costs, suggesting that financial innovation tools may not only support industrial green transformation but also indirectly contribute to public health objectives by reducing the cost of doing business.

## 5 Discussion

### 5.1 Main findings

This study systematically examines the relationships among digital transformation, green finance, and the cost of financing of pharmaceutical companies. The empirical evidence supports all five hypotheses. Digital transformation positively impacts the probability of companies being granted green certification (β = 0.079, *p* < 0.01), validating hypothesis H1. Green certification significantly promotes enterprises' acquisition of green finance support (β = 0.134, *p* < 0.01), validating hypothesis H2. Green finance significantly reduces enterprise financing costs by approximately 48.7 basis points (β = −0.487, *p* < 0.01), validating hypothesis H3. Mediation effect analysis shows that 71.1% of the total effect is realized through indirect effects, with the chain mediation effect of “digital transformation → green certification → green finance → financing costs” accounting for 34.2% of the total effect, validating hypothesis H4. Analysis of heterogeneity reveals that the decrease in financing costs due to digital transformation is 1.9 times larger for essential drug enterprises than that of non-essential drug enterprises (−0.051 vs. −0.027, *p* < 0.05), thus supporting hypothesis H5. Results of mechanism analysis show that digital transformation leads to a decrease in financing costs by reducing information asymmetry and risk premiums, which explain 39% and 53% of the total effect, respectively.

### 5.2 Public health implications

This study identifies a potential pathway through which green finance policies may contribute to improving access to healthcare by reducing financing costs for pharmaceutical companies. While the empirical evidence directly demonstrates the financing cost reduction, the subsequent transmission to drug prices and accessibility represents a theoretical implication that requires further empirical verification. The decrease in financing costs could allow more flexibility in price adjustments. Despite the fact that the degree of transmission of cost savings to the prices of terminal drugs is dependent on several factors, the special nature of essential drugs makes it relatively easy to implement a smoother transmission pathway (42). China has adopted the management of essential drugs through a price regulatory system that requires enterprises to maintain price stability and achieve reasonable profits. The reduction in financial cost relieves financial burdens on enterprises and increases their flexibility in terms of offering concessions on prices.

The financing cost reduction could potentially improve drug accessibility through three dimensions: lower patient expenses (43), expanded coverage in rural areas, and enhanced quality via R&D investment (44). These represent theoretically plausible channels that merit direct empirical testing in future research. Findings are consistent with the objectives of SDG 3 on universal health coverage, suggesting potential synergy between environmental and health equity goals (45). The green finance mechanism has special relevance for pandemic preparedness, helping maintain drug supply and price stability during public health emergencies (46).

### 5.3 Policy implications

The findings have important implications for the regulatory policies of green finance. The regulatory authorities should improve the green finance standard system, define the criteria for identifying green projects in the pharmaceutical industry, and define the requirements of environmental information disclosure. Furthermore, the regulatory authorities should include typical projects of the pharmaceutical industry related to clean production lines, waste resource use, and drug life-cycle management in the green projects (47). Improve the differentiated policy design of green finance by providing greater preferential treatment to key pharmaceutical companies by setting up special green credit quotas, offering fiscal interest subsidies, and reducing risk weights to make sure that policy funds reach exactly where they are needed in terms of public health priorities.

The policies in the pharmaceutical industry should naturally integrate the green transformation and the promotion of drug accessibility. The government should set up a linkage between the decrease of the cost of funds and the adjustment of the drug prices to guide the enterprises to pass on the cost reduction to the terminal pricing through centralized drug procurement and price negotiations (48). It is recommended to set up the bonus points of green certification in the bidding and procurement stage of essential drugs to encourage enterprises to take an active role in environmental governance. It is necessary to improve the supporting policies for enterprises in the essential drug industry in aspects of favorable tax reduction subsidies, research and development subsidies, and market access policies. Improve the digital infrastructure of the pharmaceutical industry by encouraging enterprises to adopt the Internet of Things, big data, and artificial intelligence, and to improve their ability to manage the environment and information disclosure.

A more prominent place should now be given to the role of financial innovation for improving access to medications. The healthcare sector and financial regulatory departments should now focus on improving coordination of their policies and working toward implementing green finance policies (49). It is recommended that policy processes, such as the adjustment of essential drug category, the negotiation of drug prices, and the determination of payment standards in medical insurance, consider the changes in the cost of enterprise green transformation and financing costs, which will provide policy incentives to enterprises that have received green finance support. Strengthen the mechanism for forming drug prices and, within cost-plus pricing, properly account for changes in the cost of finance, and ensure that the cost cuts achieved through green transformation can flow down to patients. Set up a mechanism for monitoring and evaluating the accessibility of drugs and conduct periodic evaluations on the effect of green finance policies on drug prices, accessibility, and quality, making adjustments to policy design accordingly.

### 5.4 Limitations and future research

This study has several limitations that need to be improved in the future. The measurement of green finance relies on green bond issuance as a binary indicator, which does not capture other forms of green financing such as green loans, green credit lines, and green development funds. Given that only 14.7% of sample firms issued green bonds during the study period, this measure may underestimate the extent of green finance utilization among pharmaceutical enterprises. Future research should explore broader green finance measures as firm-level green credit data becomes more systematically available through enhanced environmental information disclosure requirements. The sample is also limited to bond-issuing firms, which introduces potential selection bias. Firms that access bond markets may systematically differ from non-issuing firms in ways that correlate with both digital transformation intensity and financing costs. While this design is necessitated by the dependent variable construction, future research could employ broader financing cost measures, such as the weighted average cost of all interest-bearing debt, to include non-bond-issuing firms and improve external validity, particularly for understanding the effects on small and medium-sized pharmaceutical enterprises. Although it explores the effect of green finance on financing costs, it cannot directly test the change in the price of drugs, but rather makes an inference through theoretical analysis. In the future, it is necessary to test the transmission effect of the change in financing costs on the price of drugs by matching the financing cost data of the company with the data of the drug price (50). The research sample is limited to A-share listed pharmaceutical companies. This limits generalization to small and medium enterprises and non-listed companies. SMEs are very different from large companies in financing patterns, obtaining green certifications, and digital transformation prowess. Future studies should generalize their sample to explore differences in policy impacts depending on enterprise size.

The digital transformation index used in this research can be prone to a measurement error because it is calculated based on annual report texts. The reporting of digital technology in annual reporting can be driven by a number of considerations, such as corporate information disclosure strategies and reporting writing style. In future research, a number of objective measures might be incorporated to calculate a comprehensive digital transformation measure (51). The duration of the study is from 2016 to 2023. This period includes the critical stage in the evolution of the green finance policy framework in China. However, the extended impact of these policies has not been experienced yet. Future studies should include a longer time period and should use time varying coefficients. Although the chain mediation analysis is grounded in institutional sequencing and supported by robustness checks using lagged variables, the annual data frequency cannot fully establish within-year causal ordering. Higher-frequency data, such as quarterly observations, would allow more precise identification of the temporal sequence among digital transformation, green certification, and green finance. Future studies with access to such data could further strengthen the causal interpretation of the mediation pathways.

The mechanisms found in this study are primarily rooted in the context of China, and the cross-country generalizability of the findings still needs to be validated. There may be significant differences among countries in the design of green finance policies, pharmaceutical price regulation, and essential drug schemes, which could lead to differences in policy outcomes. Future studies can conduct cross-national comparative studies to explore the universality and diversity of the mechanisms through which green finance affects the accessibility of drugs (52).

## 6 Conclusion

Based on the data from Chinese A-share listed pharmaceutical manufacturing companies from 2016 to 2023, this study specifically explores the mechanism by which digital transformation affects financing costs for enterprises through green certification and green finance, and its possible effects on drug accessibility. Empirical evidence shows that digital transformation can reduce information asymmetry about environmental issues, increase the chances of obtaining green certification, and consequently reduce financing costs by about 48.7 basis points. The primary mechanism of the above effect is the mitigation of the information asymmetry problem and the risk premium, which can be achieved through two micro-mechanisms. Heterogeneity analysis shows that the policy effect is stronger in the essential drug enterprises, with the decrease of the financing cost of which is approximately 1.9 times larger than the non-essential drug enterprises. This result is influenced by the moderating effect of the public health properties of the products. The reduction in the cost of financing makes possible the adjustment of pricing in pharmaceutical enterprises. In particular, for essential drug companies, the attribute of goods related to public welfare and government price regulation may facilitate the transfer of reduced costs to final drug prices, which could contribute to the goal of health equity. This theoretical implication, while supported by institutional analysis, awaits direct empirical confirmation through studies linking financing cost data with drug price data.

Research contributions are reflected at three levels: theoretical, empirical, and policy. Theoretically, the integration of the theories of information asymmetry, signaling theory, and stakeholder theory provides a complete theoretical framework of the synergy of digital empowerment of green finance with the costs of finance. This provides a theoretical expansion of the current studies on micro-level green finance. Empirically, using chain mediation effect models and multiple endogeneity control methods, the three transmission pathways through which digital transformation affects financing costs and their relative contributions are quantified. This research provides empirical insights for the transmission channel of “digitalization → greening → financialization → cost reduction,” and confirms the heterogeneous roles in core drug enterprises. This provides important guidance for public health policy. In terms of policy, research findings provide a financial innovation pathway for promoting pharmaceutical industry green transformation, reducing drug prices, and improving drug accessibility. The evidence suggests that green finance policies can address environmental concerns while potentially contributing to public health objectives by reducing enterprise financing costs. This points to possible policy tools for pursuing the dual goals of environmental sustainability and health equity envisioned in the Sustainable Development Goals (SDGs), although direct evidence on health outcomes remains to be established. Regulatory authorities should improve the system of green finance standards and strengthen the idea of differentiated policy design; pharmaceutical industry departments should set up a linkage system for financing costs and drug prices; and public health departments should improve cross-departmental coordination for a coordinated promotion of green finance policies for the public health sector, so that the fruits of financial innovation benefit the groups most in need.

## Data Availability

The original contributions presented in the study are included in the article/Supplementary material, further inquiries can be directed to the corresponding author.
