# Supplementary material for: Digital transformation, green finance, and pharmaceutical affordability in China: a health economics perspective
Source: Front Public Health. 2026 May 15;14:1793373. doi: 10.3389/fpubh.2026.1793373 (PMC13219370; doi:10.3389/fpubh.2026.1793373)
Supplement: Supplementary file 1 [file Supplementary_file_1.docx]

**Appendix A: Digital Transformation Keyword Dictionary**

The digital transformation index is constructed based on keyword frequency analysis of annual reports. Keywords are organized into two categories based on the framework established in prior literature (34), with adaptations for the pharmaceutical industry.

**Category 1: Underlying Technology (16 keywords)** Artificial intelligence, machine learning, deep learning, big data, data mining, cloud computing, cloud platform, blockchain, Internet of Things, biometric recognition, natural language processing, edge computing, digital twin, virtual reality, augmented reality, 5G technology

**Category 2: Technology Application (16 keywords)** Intelligent manufacturing, smart factory, industrial internet, digital management, digital marketing, digital platform, intelligent logistics, smart supply chain, precision medicine, intelligent quality control, automated production, digital healthcare, online diagnosis, pharmaceutical traceability, intelligent warehousing, digital operations

**Construction procedure:** (1) Annual report PDF files were downloaded from Cninfo (<http://www.cninfo.com.cn>) for all sample firms from 2016-2023. (2) Text extraction was performed using Python (pdfplumber library), with preprocessing including removal of headers, footers, page numbers, and table contents to avoid counting non-narrative text. (3) Chinese word segmentation was conducted using the Jieba library, with the keyword dictionary added as a custom dictionary to ensure accurate identification. (4) For each firm-year, the total frequency of all 32 keywords was counted. (5) The digital transformation index was calculated as DT = ln(1 + total keyword frequency).
